# Eating disorders, body image dissatisfaction and their association with gluten-free diet adherence among patients with celiac disease

**DOI:** 10.1186/s40795-024-00910-5

**Published:** 2024-07-18

**Authors:** Reyhaneh Rabiee, Reza Mahdavi, Masood Shirmohammadi, Zeinab Nikniaz

**Affiliations:** 1 Student Research Committee, School of Nutrition and Food Science, Tabriz University of Medical Sciences, Tabriz, Iran; 2 Nutrition Research Center, Tabriz University of Medical Sciences, Tabriz, Iran; 3 Liver and Gastrointestinal Diseases Research Center, Tabriz University of Medical Sciences, Tabriz, Iran

**Keywords:** Celiac disease, Eating disorders, Body dissatisfaction, Body distortion, Gluten-free diet, Auto-immune disease, Genetic-predisposition, Cross-sectional

## Abstract

**Background:**

Considering the higher prevalence of psychological problems in patients with Celiac disease (CD), the current study aims to assess the prevalence of eating disorders (EDs) and body image disturbance in patients with CD and examine the possible correlation between EDs, body image dissatisfaction and distortion, and gluten-free diet (GFD) adherence in these patients.

**Methods:**

In this cross-sectional study, 217 patients with CD (18–55 years old) were recruited randomly from the CD registry database. EDs and body image issues were assessed using the 26-item Eating Attitude Test (EAT-26) and Stunkard Figure Rating Scale (FRS), respectively. Adherence to GFD was evaluated by the Celiac Dietary Adherence Test (CDAT) questionnaire.

**Results:**

The prevalence of EDs was 43.5%. Furthermore, the prevalence of body dissatisfaction and distortion was 65.9% and 41.1%, respectively. The logistic regression demonstrated a significant negative association between adherence to the GFD and EDs (OR = 2.09, 95% CI: 1.11–3.91, *P* = 0.022). However, there was no significant association between following GFD and body image dissatisfaction (OR = 1.70, CI: 0.92–3.17, *P* = 0.090), and distortion (OR = 0.65, CI: 0.36–1.18, *P* = 0.163).

**Conclusion:**

Considering the high prevalence of EDs in patients with CD and owing to the inverse association between EDs and GFD adherence, nutritionists should consider the psychological barriers in adhering to a GFD when consulting patients with CD.

**Supplementary Information:**

The online version contains supplementary material available at 10.1186/s40795-024-00910-5.

## Highlights

we determined the association between eating disorders (EDs), body image dissatisfaction with adherence to gluten free diet (GFD) in patients with celiac disease.

The prevalence of EDs was 43.5%.

The prevalence of body image dissatisfaction and distortion was 65.9% and 41.1%, respectively.

We observed a significant negative association between adherence to the GFD and EDs.

There was no significant association between following the GFD with body image dissatisfaction and distortion.

**Supplementary Information:**

The online version contains supplementary material available at 10.1186/s40795-024-00910-5.

## Introduction

Celiac disease (CD) is an autoimmune inflammatory disorder of the small intestine caused by the ingestion of dietary proteins of wheat, barley, and rye in genetically predisposed individuals [1]. The global prevalence of CD is approximately 1%, with similar rates reported in Iran [2]. The only available treatment for CD is strict adherence to a gluten-free diet (GFD) [3].

Eating disorders (EDs) are a group of psychiatric disorders characterized by abnormal or disturbed eating habits that adversely affect physical and mental health [4]. It includes binge-eating disorder, avoidant/restrictive food intake disorder, bulimia nervosa (BN), and anorexia nervosa (AN) [5]. AN is characterized by very low body weight, fear of gaining weight, and body image disturbance. People who suffer from this condition do things to make themselves lose weight or keep at a low weight. They think too much about their self-worth based on how much they weigh and how they look [6]. Among mental health disorders, EDs have one of the highest mortality rates and is related to morbidity for sufferers [7]. The prevalence of EDs in Asia ranges from 4.1% in Israel to 40.5% in Jordan [8]. Previous studies have suggested that certain conditions, such as autoimmune diseases, may increase the risk of developing EDs. As such, a potential association between eating pathology and CD has been proposed [9]. Since CD requires strict adherence to a GFD, this may lead to food restriction, which can potentially distort attitudes toward eating and weight and increase the risk of developing EDs [10]. However, studies investigating the association between adherence to GFD and EDs have reported mixed results [11], One study found a negative association between increased adherence to a GFD and the likelihood of developing EDs [12] However, another study reported that celiac patients who adhered to a GFD had a higher risk of developing EDs [13].

In addition to EDs, previous studies have shown that patients with chronic diseases have higher body dissatisfaction than healthy individuals [14]. Patients with CD have also been shown to exhibit higher levels of concern about shape, weight, and appearance compared to the general population [13]. However, studies examining the association between adherence to GFD and body image issues have reported inconsistent findings, one study showed increasing concern about weight and appearance in women with CD as they closely follow the GFD [13]. Although, another study has shown that GFD adherence improved body image in patients suffering from irritable bowel syndrome (IBS) [15].

Given the importance of adhering to a GFD in patients with CD, it is crucial to identify factors that affect adherence. However, there have been limited studies investigating the association between GFD adherence and eating disorders EDs and body image issues in patients with CD, and the results have been controversial [12, 13, 15]. Therefore, we aim to assess the prevalence of EDs and body image dissatisfaction and distortion (the difference between perceived current and actual image) and also examine the possible association between EDs, body image dissatisfaction, and body image distortion with GFD adherence in patients with CD.

## Material and method

### Study population and ethics aspects

In the current cross-sectional study conducted from September to December 2022, patients with CD, registered at the East-Azerbaijan CD registry of Emam Reza hospital until December 2022, were selected by convenient sampling. The CD registry in East Azerbaijan was established in 2016 as part of the national CD registry in Iran, to register key demographic, clinical, and laboratory data of patients with CD. Currently, the registry contains records for 464 participants. All patients were diagnosed based on intestinal biopsies and serology tests by gastrointestinal specialists. Histologic evaluation of the small intestinal mucosa by endoscopic biopsy samples is the gold standard for the diagnosis of celiac disease. Marsh classification is a histological classification system in patients with CD. Based on this classification, Marsh I described increased numbers of intraepithelial lymphocytes with normal mucosal and villus architecture, Marsh II described hyperplastic crypts and increased crypt cell division, Marsh IIIa described hyperplastic crypts and partial villus atrophy, Marsh IIIb described hyperplastic crypts and subtotal villus atrophy, and Marsh IIIc described hyperplastic crypts and total villus atrophy. In the present study, the patients with biopsy-confirmed CD were included if they were aged 18–55 years old. Pregnant and lactating women, patients with untreated comorbidities including diabetes, and thyroid diseases were excluded.

The study protocol was approved by the Ethics Committee of Tabriz University of Medical Sciences, Tabriz, Iran (ethical code: IR.TBZMED.REC.1401.582). All subjects were made aware of the content of the study, and informed consent was obtained. An expert dietian completed the questionnaires by face-to-face interviews in Imam Reza hospital. All variables were evaluated by one person to prevent measurement errors.

### Sample size calculation

The sample size of the study was calculated based on the result of the previous study [13] and considering the confidence interval of 95% and the error of 0.03. Then based on the following formula (N: 300, P: 22, d: 0.03) the calculated sample size was 175 patients. Considering the drop-out rate of 20%, 217 patients were recruited.

$${\rm{N}}\;{\rm{ = }}\;{{{\rm{N}}\;{{\rm{Z}}_{{\rm{1-\alpha/2}}}}^{\rm{2}}{\rm{p(1 - p)}}} \over {{{\rm{d}}^{\rm{2}}}{\rm{(N - 1)}}\;{\rm{ + }}\;{{\rm{Z}}_{{\rm{1-\alpha/2}}}}^{\rm{2}}{\rm{p(1 - p)}}}}$$

### Eating disorders assessment

The Eating Attitude Test-26 (EAT-26) was used to determine the risk for EDs in the participants by using standardized measures of eating attitudes and behaviors. The EAT-26 is a screening tool designed to identify behaviors and attitudes that indicate possible EDs. Three different disordered eating behaviors can be identified based on the responses to each item. These behaviors are reflected in three subscales: attitudes relative to dieting (questions 1, 6, 7, 10, 11, 12, 14, 16, 17, 22, 23, 24, 26), bulimia and food preoccupation (questions 3, 4, 9, 18, 21, 25), and food oral control (questions 2, 5, 8, 13, 15, 20) [16]. The value of each item is 0 to 3 and the total score is between 0 and 78. A score of 20 or more indicated a high risk of EDs [17]. The Persian version of the questionnaire was validated by Gargari et al. in Iranian population [18].

### Body image assessment

We assessed body image perception using the Figure Rating Scale (FRS), which asks participants to select the figure that best represents their current body shape and their ideal body shape. The FRS consists of nine figures ranging from very thin (underweight) to very obese (morbidly obese) [19, 20] (Fig. S1). Based on other studies, these figures were categorized into underweight (Fig. S1 number. 1 and 2), normal weight (Fig. S1 number. 3 and 4), overweight (Fig. S1 number. 5 through 7) and obese (Fig. S1 number. 8 and 9) [21]. A previous study indicated good test-retest reliability and acceptable validity [22]. Body dissatisfaction was calculated based on the differences between the perceived current image and the ideal image. Moreover, body image distortion was calculated as the difference between the perceived current and the real image. Positive and negative scores of body image dissatisfaction in this study demonstrated that the participants are dissatisfied with being heavier than ideal and lighter than ideal respectively. A score of zero was designated as indicating body shape satisfaction. The positive and negative scores of body image distortion were representing that the participants overestimate and underestimate their current sizes, respectively.

Weight (kg) and height (cm) were measured using a Seca digital weighing scale and a stadiometer, respectively. Weight and height of patients were measured with clothing and shoes removed, in order to obtain accurate measurements. The, body mass index (BMI) was calculated. For the purposes of this study, the World Health Organization’s (WHO) definitions were used to categorize participants based on their BMI. Underweight was defined as a BMI < 18 kg/m2, normal weight as a BMI of 18-24.99, overweight as a BMI of 25-29.99, and obesity as a BMI ≥ 30 was used.

### Celiac dietary adherence test

To assess adherence to a gluten-free diet (GFD), we used the Persian version of the CD Adherence Test (pv-CDAT), a validated questionnaire that assesses the level of adherence to a GFD [23]. The CDAT is a 7-item instrument, rated via Likert scale (0–5) from never to always, designed and developed to assess the level of adherence to a GFD. A high correlation exists between the result of the CDAT and standardized dietitian evaluation and IgA-TTG [24]. It includes questions about symptoms, self-efficacy, and gluten avoidance habits. The questionnaire score ranges from 7 to 35. The higher score demonstrates poorer adherence [24]. A score of ≥ 13 was considered a cut-off to display non-adherence. An expert dietitian completed CDAT questionnaire by face to face interview.

### Statistical analysis

The data were analyzed using SPSS software (version 23) [25]. We used the Kolmogorov-Smirnov test to assess normal distribution, and reported means and standard deviations (SD) or medians and 25th and 75th percentiles for normally and non-normally distributed continuous variables, respectively. Frequencies and percentages were calculated for nominal and ordinal variables. We used independent sample t-tests (for normally distributed variables) and Mann-Whitney U tests (for non-normally distributed variables) to compare continuous variables between groups, and chi-square tests to compare nominal and categorical variables. We used logistic regression to assess the association between compliance with a gluten-free diet (GFD) as independent variables and eating disorders (EDs), body image dissatisfaction, and body image distortion as the response variables. We considered three models: model 1 (unadjusted), model 2 (adjusted for demographic variables such as age, sex, and BMI), and model 3 (further adjusted for clinical factors including symptoms, comorbidities, disease duration, and years following a gluten-free diet). We considered a p-value of < 0.05 as significant.

## Results

**Fig. 1:**
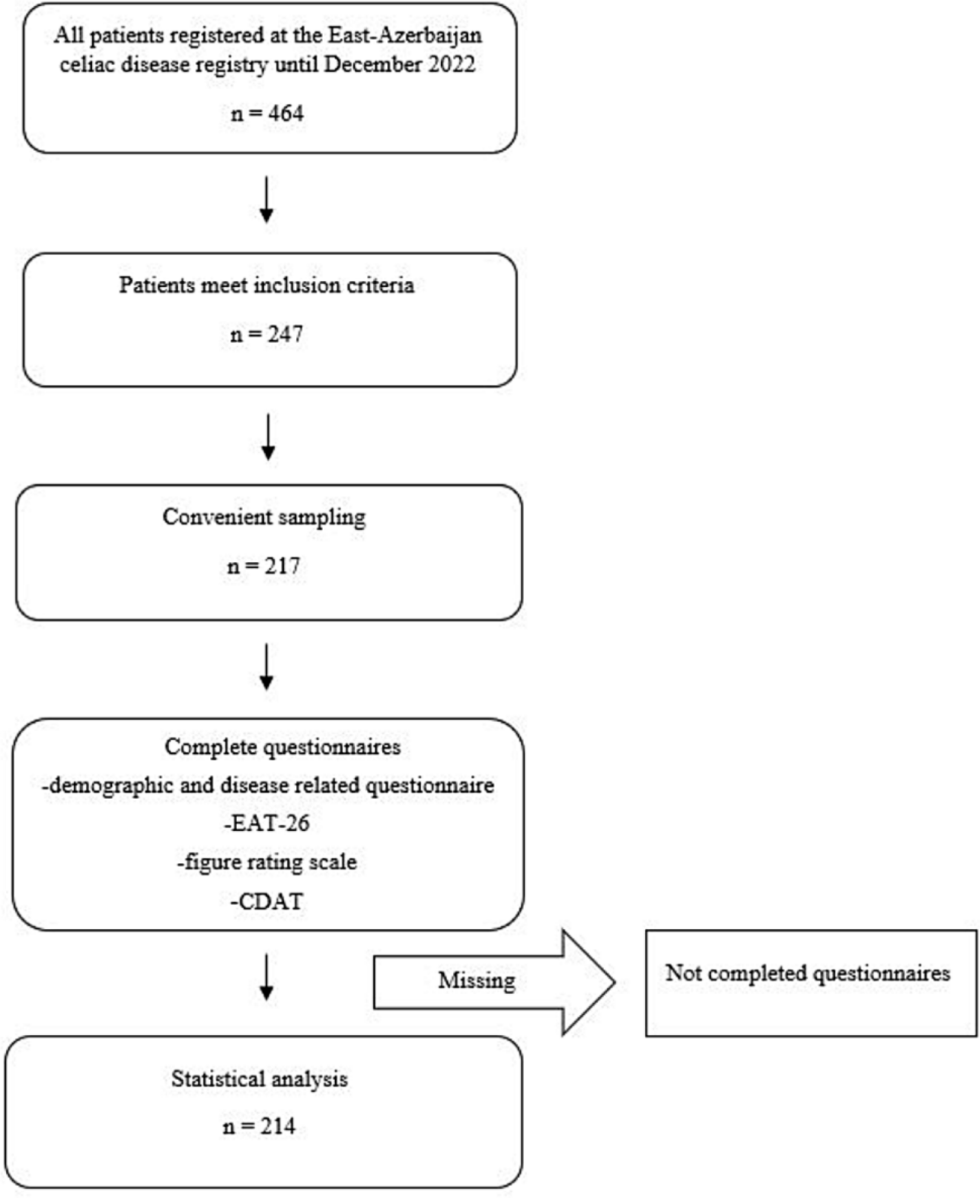
Flow chart of patients’ recruitment and analysis CDAT: Celiac Disease Adherence Test; EAT-26: EAT-26, Eating Attitude Test-26; GFD, gluten-free diet

Of 464 CD patients registered at the East Azarbijan CD registry, 247 met the inclusion criteria. Finally, 217 patients with CD were entered by convenient sampling. Three patients were excluded from the final analysis because they did not complete all questionnaires. There were no statistically significant differences in the general characteristics of the excluded patients and the remaining 214 participants. The flow chart of patient recruitment and analysis is illustrated in Fig. 1.

Table 1 shows the demographic and clinical characteristics of patients grouped by GFD adherence status. The mean age of participants was 38.55 ± 9.65 years, and 58.9% of them were female. The mean BMI was 24.27 ± 4.15 kg/m2, and 56.1% had normal BMI levels. Additionally, 59.8% of patients were non-adherent to GFD. Patients with non-adherence to GFD had significantly higher manifestations of symptoms (*P* = 0.003) compared to the adherence group, but there were no statistically significant differences between the two groups regarding other variables.

**Table 1:** Descriptive demographic and clinical data of study participants

	Total	Adherence to GFD (CDAT < 13) *n* = 86	Non-adherence to GFD (CDAT ≥ 13) *n* = 128	*P*-value
Age (years) (mean ± SD)	38.55 ± 9.65	38.89 ± 10.31	38.32 ± 9.21	0.670
Sex n% Male Female	88(41.1%) 126(58.9%)	43(49.4%) 44(50.6%)	46(35.4%) 84(64.6%)	0.060
Weight (Kg) (mean ± SD)	65.84 ± 12.75	66.94 ± 10.98	65.10 ± 13.81	0.280
BMI (Kg/m^2^) (mean ± SD)	24.28 ± 4.17	24.24 ± 3.48	24.31 ± 4.59	0.905
BMI categories n% < 18.5 (underweight) 18.5-24.99 (normal) 25-29.99 (overweight) ≥ 30 (obesity)	11(5.1%) 120(56.1%) 61(28.5%) 22(10.3%)	3(3.5%) 51(59.3%) 25(29.1%) 7(8.1%)	8(6.3%) 69(53.9%) 36(27.7%) 15(11.7%)	0.640
Duration on GFD (years) Median [IQR]	5 (2, 8)	5 (3, 8)	5 [1,7.25]	0.068
disease Duration (years) Median [IQR]	6 (3, 8)	6 (3, 9)	6 (3, 8)	0.860
Marsh n% I II IIIa IIIb IIIc	21(9.8%) 29(13.6%) 57(26.6%) 76(35.5%) 31(14.5%)	7(8.1%) 12(14%) 26(30.2%) 28(32.6%) 13(15.1%)	14(10.9%) 17(13.3%) 31(24.2%) 48(37.5%) 18(14.1%)	0.823
Having symptom n%	133(62.1%)	43(50%)	90(70.3%)	0.003
Having Other disease n%	105(49.1%)	41(47.7%)	64(50%)	0.739
Drug use n%	99(46.3%)	41(47.7%)	58(45.3%)	0.734
Supplements use n%	137(64%)	54(62.8%)	83(64.8%)	0.759

BMI, body mass index; CDAT, Celiac Disease Adherence Test; GFD, gluten-free diet

**Fig. 2:**
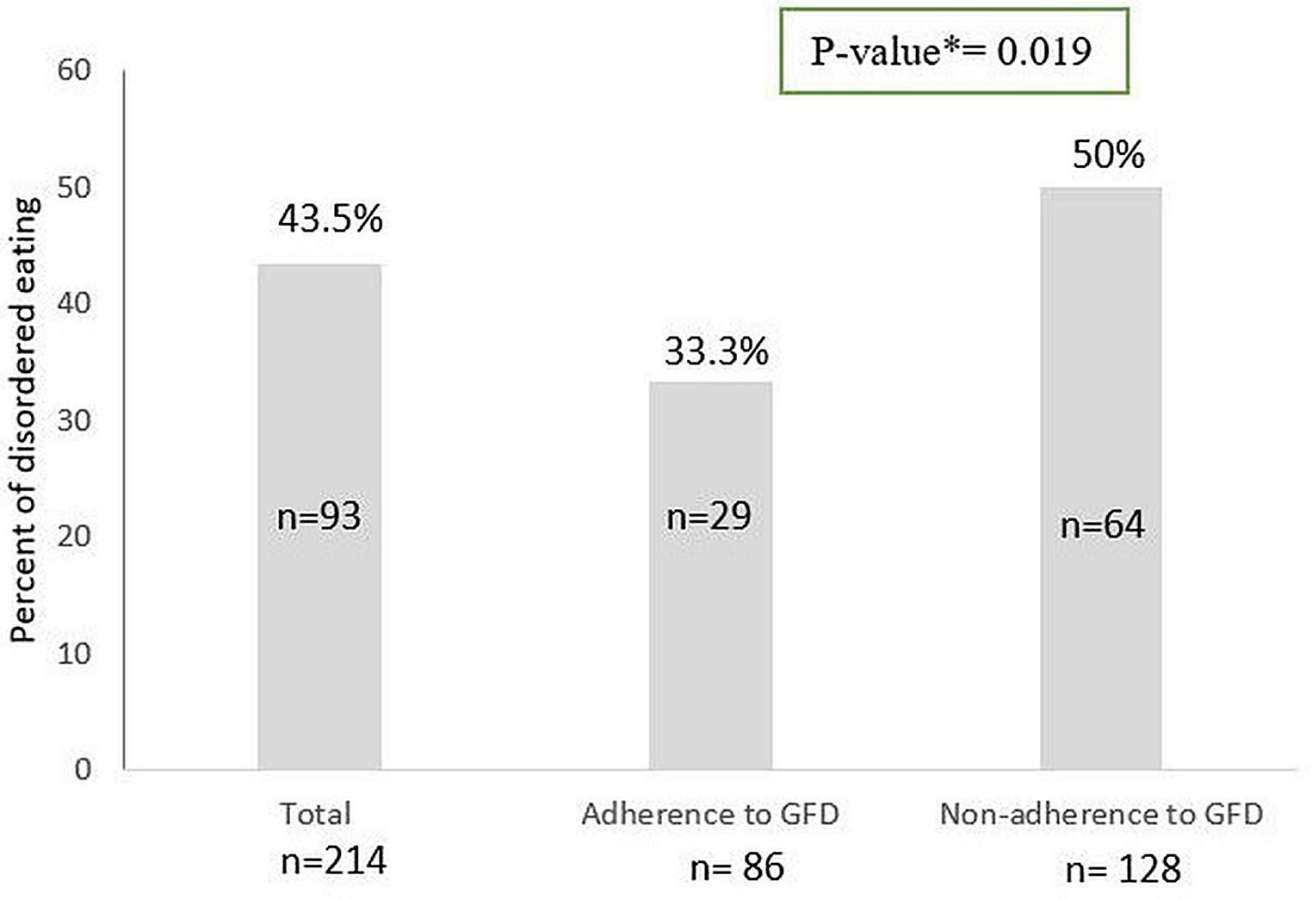
Percentages and number of pathological EAT-26 scores in total individuals, adherence and non-adherence to GFD with CD CD, celiac disease; EAT-26, Eating Attitude Test-26; GFD, gluten-free diet *p-value (Ch Squair)

Figure 2 illustrates the percentage of CD patients with EDs stratified by adherence level. In total, 43.5% of patients had EDs, and participants with non-adherence to GFD (50%) had a significantly higher prevalence of EDs compared to the adherence group (33.3%) (*P* = 0.019).

As presented in Table 2, the mean score of bulimia was significantly higher in the GFD non-adherence group compared to the adherence group (*P* = 0.007). In addition, non-adherence to GFD had a significantly higher mean score of oral control than adherence group (*P* = 0.002). However, no significant difference was demonstrated in the mean score of the dieting scale between the two groups (*P* = 0.921).

**Table 2:** Descriptive statistics presented as mean-standard deviation for EAT-26 subscale scores

	Adherence to GFD (CDAT < 13) *n* = 86	Non-adherence to GFD (CDAT ≥ 13) *n* = 128	*P*-value*
Subscale of EAT-26 (mean ± SD)			
Dieting scale	8.59 ± 4.30	8.53 ± 5.20	0.921
Bulimia	3.33 ± 2.76	4.46 ± 3.33	0.007
Oral control	5 ± 3.09	7.17 ± 4.93	0.002

CDAT, Celiac Disease Adherence Test; GFD, gluten-free diet; EAT-26, Eating Attitude Test-26

*Independent sample t-test

**Fig. 3:**
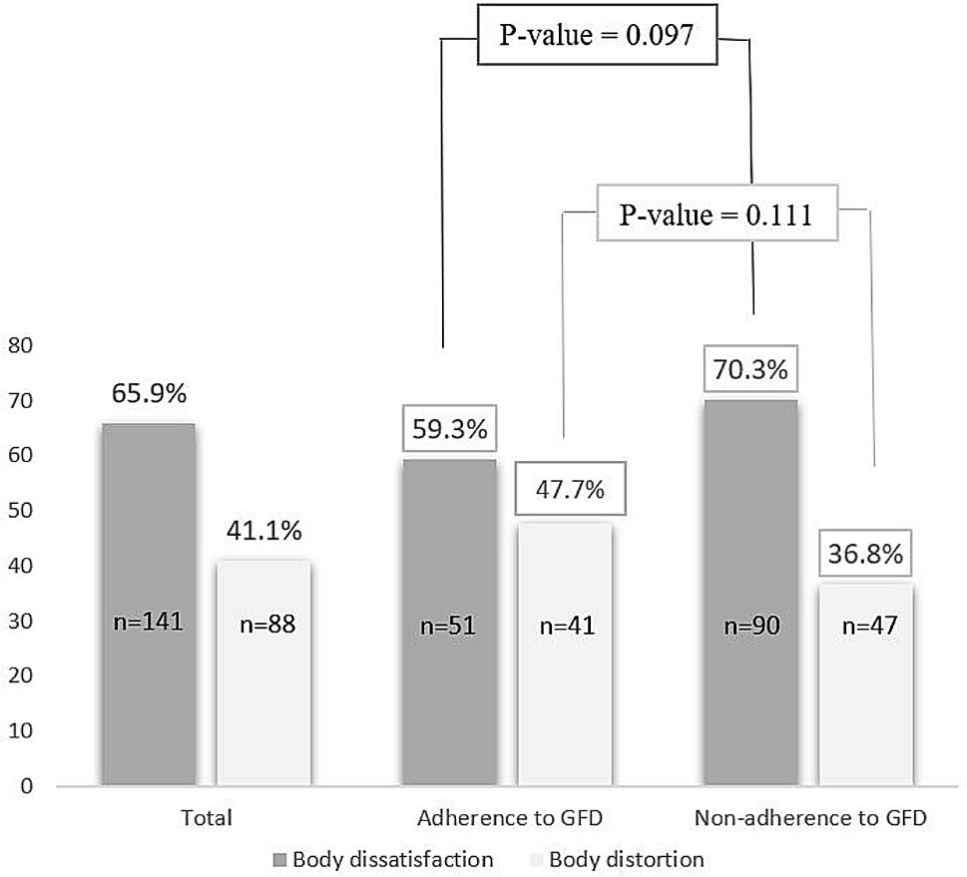
Percentages and number of eating body dissatisfaction and distortion in total individuals, adherence and non-adherence to GFD with CD CD, celiac disease; GFD, gluten-free diet *p-value (Chi-Square)

Figure 3 shows the prevalence of body dissatisfaction and body distortion in patients with CD. In total, 65.9% of patients had body image dissatisfaction, and 41.1% of them had body image distortion. No statistically significant differences were observed in body image dissatisfaction (*P* = 0.097) and distortion (*P* = 0.111) between GFD adherents and non-adherents.

Table 3 shows the regression analysis for the association between adherence to GFD and EDs, body image dissatisfaction, and distortion. GFD non-adherence patients had a higher chance of having EDs in all crude and adjusted models (OR = 2.09, 95% CI: 1.11, 3.91 for the last model). However, no significant statistical correlation between adherence to GFD and body image dissatisfaction and distortion was found.

**Table 3:** Crude and adjusted OR of the non-adherence to GFD in patients with CD

	Odd ratio (95%CI)	*P*-value*
Eating disorder (^a^ Ref = no) Crud model Model 1** Model 2***	1.96 (1.11–3.46) 1.89 (1.06, 3.37) 2.09 (1.11, 3.91)	0.019 0.030 0.022
Body dissatisfaction (^a^ Ref = no) Crud model Model 1 Model 2	1.62 (0.91–2.88) 1.75 (0.97, 3.14) 1.70 (0.92, 3.17)	0.097 0.061 0.090
Body distortion (^a^ Ref = no) Crud model Model 1 Model 2	0.63 (0.36–1.11) 0.66 (0.37, 1.17) 0.65 (0.36, 1.18)	0.111 0.159 0.163

Non adherence to GFD was considered as independent variable

*Logistic regression models were used to estimate odd ratio with 95% CIs.

**Model I: adjusted for age, gender, and body mass index

***Model II: adjusted for age, gender, symptoms, comorbidities, disease duration, and duration on GFD

^a^ Ref: reference group

GFD, gluten-free diet; CI, confidence intervals

## Discussion

Patients with CD are required to follow lifelong dietary recommendations, which may be associated with psychological problems. In a cross-sectional study, we found that the prevalence of eating disorders (EDs) among CD patients was 43.5%. This is consistent with a previous study that reported a prevalence of 42.1% among Iranian CD patients [26]. Another study found that over 48% of CD patients were at risk of avoidant/restrictive food intake disorder [27]. Compared to the general population, the prevalence of EDs was significantly higher in CD patients. While the prevalence of EDs in Iran was 22%, it has not been reported in East Azerbaijan [28]. In contrast, studies conducted in developed countries showed a lower prevalence of EDs. For instance, Satherley et al. reported a prevalence of 19.1% in British celiac patients [29], and the prevalence of EDs was 29.3% in Australian patients [30]. The higher prevalence of EDs in our study may be attributed to differences in sex ratio, disease duration, food cultures, and assessment tools. Moreover, the COVID-19 pandemic may have contributed to the higher prevalence of EDs observed in this study. Previous studies have suggested that the pandemic may have led to increased stress, depression, changes in physical activity, and eating habits [31, 32]. Tavalacci et al. reported that the prevalence of EDs significantly increased from 31.8% to 13% in 2018 to 51.8% and 31.3% in 2021 for women and men, respectively [33]. In addition, fear of COVID-19 was significantly correlated with a higher risk of EDs [34].

We also found an inverse association between adherence to a gluten-free diet (GFD) and the prevalence of EDs, which is consistent with previous studies [12, 29, 35]. However, some studies reported no association or positive association between GFD adherence and EDs in celiac patients [11, 13, 36, 37]. The discrepancy between different studies may be due to differences in age, sex proportion, methods of body image and GFD adherence assessment, and the number of years on GFD. The exact mechanism that explains the relationship between GFD compliance and EDs is still unclear. It is possible that following GFD may decrease anxiety levels, increase general psychological functioning, and positively affect eating behaviors [38]. Besides, the belief that a dietary regimen is associated with weight gain may lead to non-adherence to GFD and consumption of trigger foods for weight loss. These eating patterns may cause the criteria for eating pathology and can span the spectrum of disordered eating behaviors [39]. Additionally, non-adherence to GFD may be associated with nutritional deficiency, which may initiate EDs [40]. For example, iron deficiency may impair the function of neurotransmitters such as serotonin, dopamine, and norepinephrine and result in EDs [41, 42]. Following GFD can improve intestinal villi, helping to absorb nutrients better and reducing EDs [3].

Our study is the first to examine the prevalence of body image dissatisfaction and distortion in patients with CD. We found a prevalence of 65.9% and 41.1%, respectively, which is consistent with previous research on autoimmune diseases. The prevalence of body dissatisfaction was reported to be 70% and 54.5% in patients with inflammatory bowel disease and type I diabetes respectively [43, 44]. Body image disturbances in CD may result from symptoms such as weight loss, abdominal pain, diarrhea, fatigue, osteoarticular, and dermatologic symptoms. In addition, a positive relationship between conditions associated with increasing inflammatory factors like C-reactive protein (CRP) and tumor necrosis factor-alpha (TNF-α) with body dissatisfaction has been shown [45, 46]. However, we found no significant relationship between body dissatisfaction and body distortion with adherence to GFD, which conflicts with previous findings. In patients with CD, it has been shown increasing concern about shape, weight, and appearance with increasing adherence to GFD in women [13]. In patients with IBS, Paduano et al. demonstrated a significant improvement in body image in patients who adhere to GFD [15]. Moreover, Compagna et al. showed that GFD significantly improves body image satisfaction in healthy subjects [47]. The discrepancies between the results may partly be due to the differences in BMI, age, sex proportion, health conditions, sample size, and adherence level to GFD.

The study was conducted by a trained dietitian interviewer who interviewed participants randomly selected from the celiac disease registry. This increases the generalizability of the findings. However, the result of the study should be interpreted cautiously condifring the limitations of the study such as the cross-sectional design of the study, which limits the ability to determine cause-and-effect relationships. Moreover, other factors such as anxiety and depression may also play a predictive role in EDs risk [48], which we were limited in considering in the relationship between EDs and adherence to a GFD. In this study, we did not use serum markers such as tTG-IgA to measure compliance with GFD, although the previous study have shown that CDAT is highly correlated with antibody levels [24]. In addition, we did not assess other barriers such as physical and economic access to gluten-free products and the psychological status of participants. Nonetheless, we believe that our findings increase the understanding of EDs and body image disturbances in patients with CD. They also highlight the importance of adhering to GFD in reducing their prevalence.

## Conclusion

The study found that 43.5% of people with celiac disease (CD) also have eating disorders (EDs). Moreover, 65.9% of CD patients suffer from body image dissatisfaction, and 41.1% experience body image distortion. On the other hand, following a gluten-free diet (GFD) was found to have a negative association with the development of EDs. Therefore, it is recommended that CD patients receive psychological interventions, such as cognitive or self-management therapies, to address the high prevalence of EDs and body image dissatisfaction. Also, nutritionists should be aware of the potential psychological barriers that may hinder adherence to a GFD. However, the study has certain limitations, and more longitudinal studies are needed to confirm these findings. These studies should also take into account other potential factors, such as economic status, anxiety, and depression, to gain a more comprehensive understanding of the relationship between CD, EDs, and body image issues.

### Electronic supplementary material

Below is the link to the electronic supplementary material.

Supplementary Material 1

Supplementary Material 2

## Data Availability

The data are available from the corresponding author upon request.

## Abbreviations

AN: Anorexia nervosa
BMI: Body mass index
BN: Bulimia nervosa
CD: Celiac disease
CDAT: Celiac dietary adherence test
CRP: C-reactive protein
EAT-26: Eating attitude test-26
EDs: Eating disorders
FRS: Figure rating scale
GFD: Gluten free diet
IBS: Irritable bowel syndrome
OR: Odds ratio
SD: Standard deviation
TNF-α: Tumor necrosis factor-alpha
