# Supplementary figures and images for: Eating disorders, body image dissatisfaction and their association with gluten-free diet adherence among patients with celiac disease
Source: BMC Nutr. 2024 Jul 18;10:100. doi: 10.1186/s40795-024-00910-5 (PMC11256539; doi:10.1186/s40795-024-00910-5)

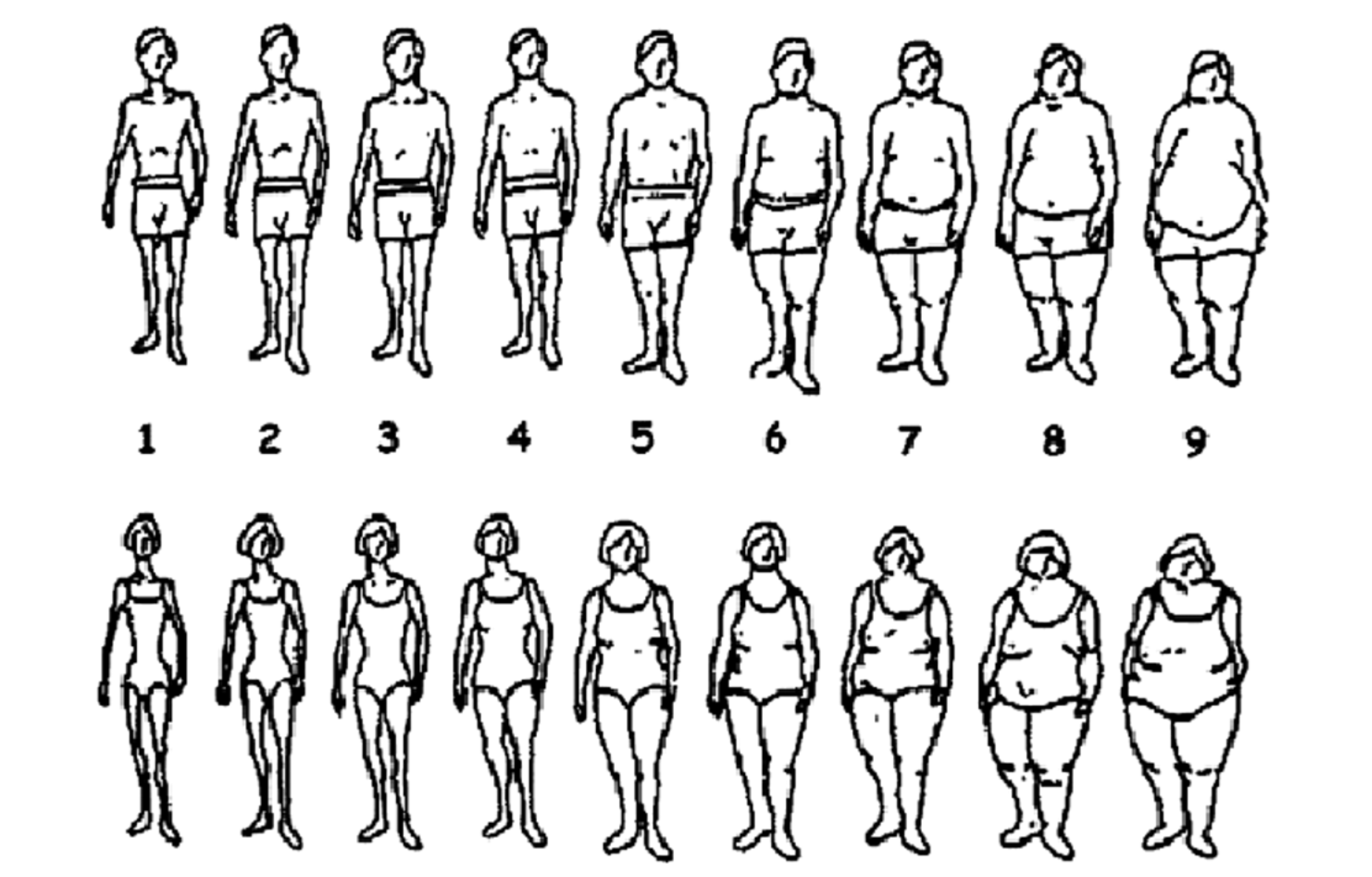

Supplement: Supplementary file 1 — Supplementary Material 1 [file 40795_2024_910_MOESM1_ESM.tiff]
